# Researching for better instructional methods using AB experiments in MOOCs: results and challenges

**DOI:** 10.1186/s41039-016-0034-4

**Published:** 2016-04-22

**Authors:** Zhongzhou Chen, Christopher Chudzicki, Daniel Palumbo, Giora Alexandron, Youn-Jeng Choi, Qian Zhou, David E. Pritchard

**Affiliations:** 1 Massachusetts Institute of Technology, room 26-321, 77 Massachusetts Avenue, Cambridge, MA 02139 USA; 2 University of Alabama , 302 Carmichael Hall Box 870231, Tuscaloosa, AL 35487 USA; 3 Qinghua University, 30 Shuang Qing Lu, Haidian, Beijing, China

**Keywords:** Cognitive Load, Deliberate Practice, Extraneous Cognitive Load, Traditional Problem, Multiple Choice Format

## Abstract

We conducted two AB experiments (treatment vs. control) in a massive open online course. The first experiment evaluates deliberate practice activities (DPAs) for developing problem solving expertise as measured by traditional physics problems. We find that a more interactive drag-and-drop format of DPA generates quicker learning than a multiple choice format but DPAs do not improve performance on solving traditional physics problems more than normal homework practice. The second experiment shows that a different video shooting setting can improve the fluency of the instructor which in turn improves the engagement of the students although it has no significant impact on the learning outcomes. These two cases demonstrate the potential of MOOC AB experiments as an open-ended research tool but also reveal limitations. We discuss the three most important challenges: wide student distribution, “open-book” nature of assessments, and large quantity and variety of data. We suggest possible methods to cope with those.

## General background

With tens of thousands of registered students, massive open online courses (MOOCs) hold great promise for data-driven education research (Ho et al. 2015; Reich 2015; Williams 2013). Research in MOOC started almost simultaneously with the launch of the first MOOCs, covering topics from student behavior (Han et al. 2013; Kizilcec et al. 2013, edX; Koedinger et al. 2015) and instructional strategies (Guo et al. 2014; Kim et al. 2014; Piech et al. 2013) to learning outcomes (Colvin et al. 2014). An important recent advance in MOOC-based education research is the capability to conduct an “AB experiment” on major MOOC platforms (Anderson et al. 2014; edX, n.d.; Williams and Williams 2013).

Also called split testing, AB testing, or randomized controlled trial, AB experiments are a research method that is used by a wide range of disciplines ranging from marketing and business intelligence to medical research and social sciences (Chalmers et al. 1981; Kohavi and Longbotham 2015). Simply put, in a MOOC AB experiment, different instructional materials are presented to different groups of students, and the effectiveness of the materials can be evaluated by measuring the behavior and learning outcomes of students using various metrics.

MOOC AB experiments can be a powerful tool to help sort through the enormous variety of instructional choices. Koedinger et al. (2013) estimated the raw number of possible instructional choices to be on the order of an impossible 3^30^, which includes decisions such as more worked examples vs. more tests, immediate vs. delayed feedback, and longer vs. shorter videos. With large number of students and automated group randomization scheme, multiple MOOC AB experiments can be easily implemented in a single course, enabling researchers to accumulate evidence on different instructional designs at a much faster rate.

However, the intrinsic “openness” of MOOCs also poses unique challenges on both the design of AB experiments and the analysis of the result. For example, unlike most clinical trials in the social sciences where the experimenter has almost complete control over the behavior of participating subjects, MOOC instructors have little to no control over the subjects. How the experiment designs and data analysis methods developed for offline experiments can be modified to suit the online environment is still a largely open question.

The RELATE group at MIT is among the first to conduct multiple AB experiments in MOOCs. In the 2014 run of our introductory physics MOOC 8.MReVx, we performed as many as seven AB experiments, investigating various aspects ranging from videos to problems to virtual labs. In this paper, we present in detail the design, implementation, analysis, and result of two of those experiments.

The first experiment evaluated a new type of training activity inspired by research on “deliberate practice” (Ericsson 2009; Ericsson et al. 1993; Ureña 2004) focusing on finding the best practice activity to develop expertise in solving physics problems. We compared them with “standard practice” as a control—training on solving multiple traditional physics problems. In addition, we also investigated whether a new interactive problem format, drag-and-drop problems, might be a more effective form of deliberate practice, as compared with a more traditionally formatted (and much easier to create) multiple choice format.

In the second experiment, we studied how different video shooting setups can affect the performance of the instructor, as well as the behavior and learning of students. While the first experiment concentrated on assessing performance and skill, the second experiment also explores the behavioral pattern of MOOC students and instructors. It is unique in the sense that in this experiment the MOOC instructor becomes the subject of investigation along with the students.

In addition to presenting the analysis and results of each experiment, we will emphasize on comparing different experiment designs and data analysis decisions and how each of them suits the MOOC environments. We will also share the challenges we face in analyzing exceptionally noisy MOOC data, as well as lessons we learned from unsuccessful attempts. With these, we hope that this paper can be of broader interest and value to the general online learning research community.

### Implementation of AB experiments on the edX platform

The edX platform has the ability to implement AB experiments (called “content experiments” by edX) (edX, n.d.) in which the user population is partitioned into two or more groups and each group is given a different version of course material. Some important aspects of edX AB experiments are as follows:

- Users are randomly assigned to two or more groups for a given experiment.
- Group assignment takes place when the user’s browser first attempts to access the experiment material, resulting in equal number of subjects per group despite a high level of random attrition among the MOOC population.
- All users participate in all experiments or opt out of the course itself.
- For each instance of the experiment, the instructor can choose to either keep using existing randomized groups or use a new random group assignment.

### Structure of 8.MReVx mechanics review MOOC

The experiments described in this paper are conducted in our MOOC 8.MReVx Introductory Mechanics, which is designed for those with some existing knowledge of Newtonian mechanics. It concentrates on problem solving and uses a pedagogy called Modeling Applied to Problem Solving (Pawl et al. 2009).

The course consists of 12 required and 2 optional weekly units. A typical unit contains three sections: instructional e-text pages (with interspersed concept questions), homework, and quiz. Most graded problems in the course allow for multiple attempts. In general, three attempts are allowed for numeric and symbolic response problems on the quiz, and up to 10 attempts are allowed in homework. The number of attempts on any multiple choice problem equals half of the available choices to discourage guessing. We had ~11,000 students who registered in the course, with just over 500 receiving certificates.

## Experiment 1: developing physics expertise using deliberate practice in drag-and-drop format

### Background

#### Developing expertise in physics

Research in educational psychology (Bransford et al. 2000; Chi et al. 1981; DeGroot 1965) has long revealed that domain experts possess a variety of skills that distinguish them from novices, such as quickly extracting important information from complex situation (Chase and Simon 1973; DeGroot 1965; Egan and Schwartz 1979; Lesgold et al. 1988), organizing knowledge around important ideas or concepts (Chi et al. 1981; Wineburg 1991), and displaying great fluency in retrieval of their knowledge (Lesgold et al. 1988; Schneider and Shiffrin 1977; Simon et al. 1980).

Yet despite extensive knowledge of expert characteristics, most introductory physics courses are still largely unsatisfactory in terms of engendering physics expertise in college physics students. College physics students frequently employ heuristic methods such as equation hunting during problem solving, rather than using more expert like strategies (Tuminaro 2004; Walsh et al. 2007).

Careful research shows that a critical factor in the development of expertise in domains from music to sports to chess is the amount of time spent on so-called deliberate practice (Ericsson et al. 1993; Ericsson 2006; Ureña 2004). Deliberate practice has three defining characteristics. First of all, each practice activity must focus on a single aspect of expertise rather than playing the “full game.” For example, tennis players separately practice their serve, backhand, and drop shot. Secondly, the same practice is repeated multiple times until a certain level of mastery is achieved. Finally, during deliberate practice, the practitioner often receives instant, directed feedback on his/her progress.

The standard approach to teaching introductory physics rests largely on having students complete lots of traditional “back of the chapter” problems—essentially “full game” practice. However, solving these problems lacks all three characteristics of deliberate practice. First of all, properly solving such a problem often involves multiple (six to seven) expert skills, yet each skill is practiced only once per problem. Secondly, the process of solving one such problem is often fairly lengthy, limiting the amount of practice on any single skill. Finally, in a traditional classroom setting, the feedback on problem solving is neither instant (often delayed from 1 day to 1 week) nor directed to any particular skill (often only on the correctness of the final answer). It is known that even after solving a significant amount of those problems, students’ physics understanding is still inadequate (Kim and Pak 2002).

#### Designing deliberate practice activities for the development of physics expertise

Following the principles of deliberate practice, we designed and developed a number of practice activities named “deliberate practice activities” (DPAs), as an alternative to practicing on traditional problems. Each DPA is consisted of a sequence of five to six problems that are designed according to the following design principles:

1. Each sequence of problems is designed to focus on one documented expert skill.
2. Each problem can be solved in a relatively short time, involving few operations irrelevant to the targeted expert skill. This allows for adequate repetitive practice in a reasonable amount of time.
3. Each problem is accompanied with detailed feedback.

**Fig. 1:**
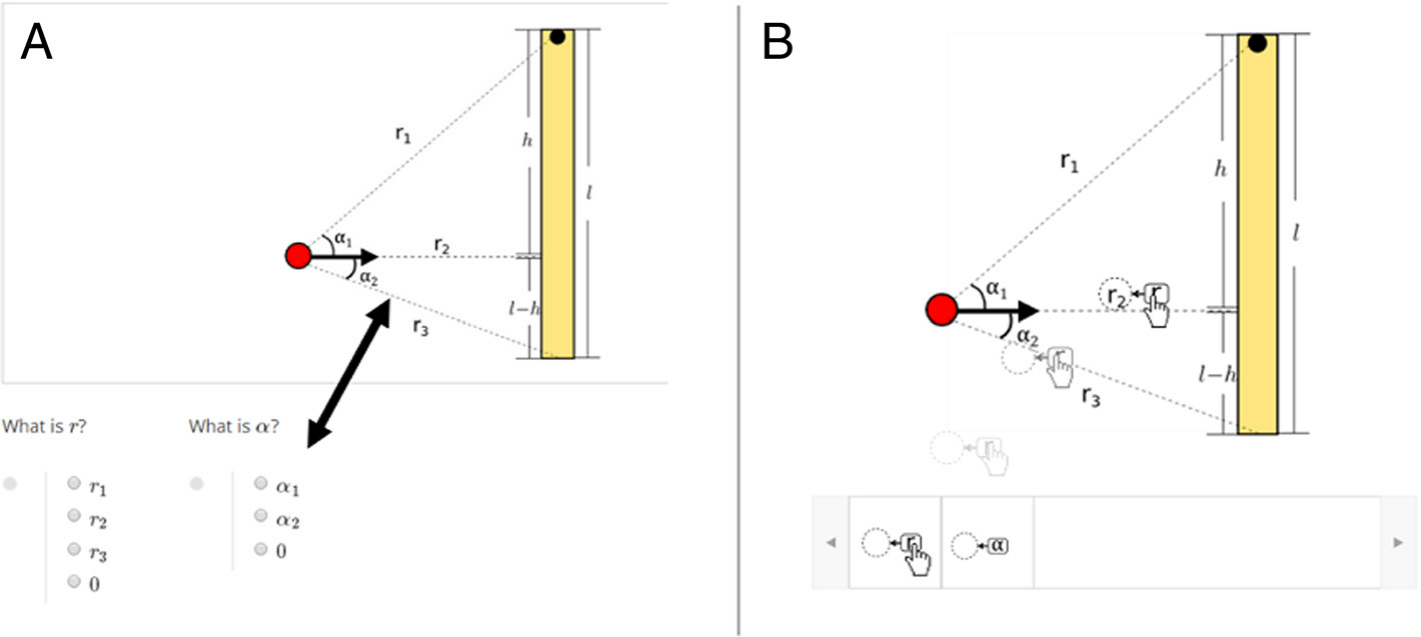
Comparison of the same problem in two formats. **a** Multiple choice format requires students to match between problem and choices. **b** Drag-and-drop format requires students to drag label to the important feature

For example, in one DPA sequence that focuses on training the expert skill of mapping between different representations, students are given the general mathematical expression of a physics situation (such as angular momentum) and required to map each variable in the expression, such as distance *r* and angle *α*, to the corresponding distances and angles in a specific given physics situation (Fig. 1).

#### Using drag-and-drop problems to improve effectiveness of DPA

A critical feature of DPA problems is that they need to be highly focused on the target skill. A good metric for “focusedness” is a lower amount of extraneous cognitive load (Gerjets and Scheiter 2004; Paas et al. 2003; Sweller et al. 2011), cognitive activities irrelevant to the skill being trained, incurred during problem solving. We observe that the traditional multiple choice (MC) problem format, including dropdown lists and checkboxes, inevitably incurs significant cognitive load, especially on DPA problems. For example, when training students to identify important features in a given situation, a multiple choice format requires students to switch back and forth between the choices and the figure, incurring much cognitive load. Previous research on problem format (Chen and Chen 2009; Gillmor et al. 2015; Huang et al. 2015) showed that students perceive higher cognitive load completing traditional multiple choice questions.

To reduce the extraneous cognitive load, we utilized a new problem format provided by the edX platform: drag-and-drop problems. “Drag-and-drop” (D&D) problems, as shown in Fig. 1b, allow students to drag a visual icon from a list to a target diagram. In this example, students indicate the important feature by directly dragging the pointer to the relevant feature.

We further reduce extraneous cognitive load in the D&D format by following multimedia design principles outlined in the study by Chen and Gladding (2014) and Schnotz (2002). In short, the theories predict that extraneous cognitive load can be further reduced when verbal/symbolic icons are replaced by visual/perceptual icons. For example, as shown in Fig. 3, angular accelerations in different directions are represented by rotation icons in different directions, rather than text such as “clockwise” or “counter-clockwise.” An additional benefit of such a design is that students’ final answer is a figure representing the physics process, which is more valuable to remember than a selected item such as item A. Utilizing multiple modes of information coding has also been shown to facilitate understanding and memorization (Mayer 2001; Schnotz and Kürschner 2008).

### Research questions

In summary, this study is designed to answer two related research questions:

1. Can DPA serve as a more effective method in developing physics expertise compared with solving traditional end-of-chapter physics problems?
2. Is the D&D format more effective in developing individual expert skill compared to traditional multiple choice format?

### Balanced experimental design and sample

#### Experimental design

**Fig. 2:**
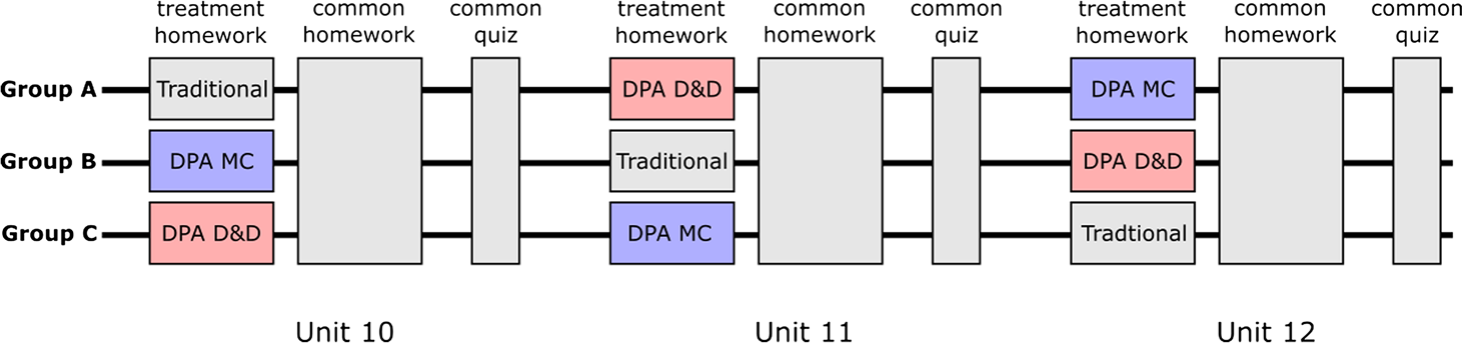
Experimental design for experiment 1

The experiments were conducted in the homework and quiz of the last three required units (10, 11, and 12) of the MOOC (Fig. 2). Students were partitioned into three groups (A, B, or C). Each group received one of three different homework treatments in each unit: DPA activities in either D&D format (DPA-D&D), MC format (DPA-MC), or traditional end-of-chapter homework (TRD). The treatments were rotated between the three groups over three units. A common second homework consisting of traditional homework problems followed the experimental treatment, unavoidably covering other topics taught in the unit that are not covered in the treatment homework. Physics problem solving expertise was assessed by a common quiz given to students together with both homework assignments. The quizzes also consisted entirely of traditional problems (mostly numeric/symbolic response).

The DPA treatment for each unit consisted of four to five DPA sequences, where each sequence consisted of three to seven DPA problems. In each unit, the first three to four DPA sequences focused on training a single, basic level expert skill. The last DPA sequence trained higher level solution planning skills by asking students to identify a number of different errors in a given solution. Each DPA treatment consisted of ~20 problems in total. Among all the DPA sequences created, five of them consisted of more than four problems each (two in unit 10, two in unit 11, and one in unit 12; these are suitable for studying students’ development of each individual skill). The TRD treatment consists of a mix of traditional symbolic problems and conceptual problems, with a total of six to ten problems each unit. Detailed solution for each problem was made available to students immediately after they have finished the problem (answered correctly or used up all the attempts). Although the deliberate practice treatment contains many more problems than the traditional treatment, both treatments cover the target material and are intended to take roughly the same amount of time.

#### DPA problems in D&D and MC format

**Fig. 3:**
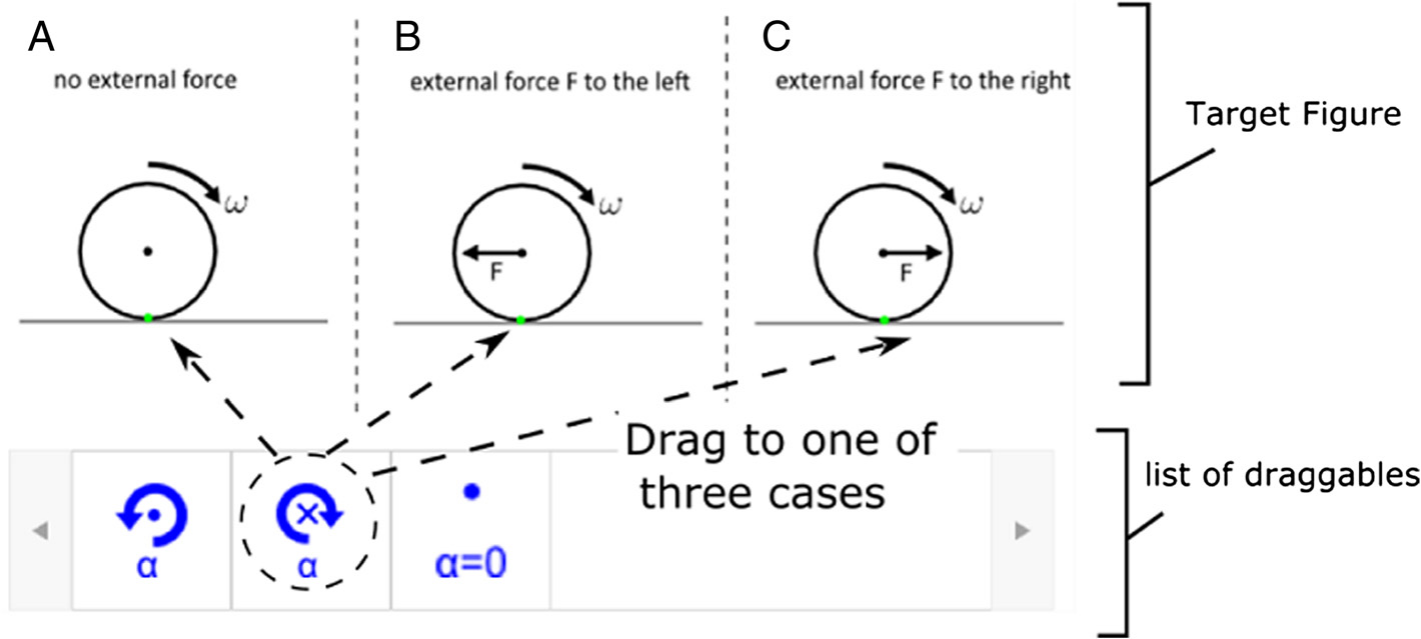
Example of drag-and-drop problem in edX. **a** No external force. **b** External force *F* to the left. **c** External force *F* to the right

An edX D&D problem (Fig. 3) consists of a target figure and a list of draggable icons called “draggables.” For each draggable, the author defines a designated target area on the target figure (different target areas can overlap each other). The problem is graded as correct when all the draggables are dropped onto their designated target areas.

Multiple attempts (5–10) are allowed for each D&D problem. A detailed solution is provided for each DPA problem involved in this experiment and is made available to students immediately after they have answered correctly or used up all their attempts.

The MC version of DPA problems involved multiple choice, checkboxes, and dropdown lists and was designed to mimic their D&D counterparts as closely as possible by using the same figures, problem text, and solution. Since D&D problems often have a very large number of possible answers, the corresponding MC version is broken into two MC sub-questions graded simultaneously, to avoid presenting too many choices. This difference theoretically gives the MC group a small advantage in problem solving, as students are informed whether their answer to each sub-question is correct after each attempt. As a result, fewer attempts are allowed for MC problems than D&D problems, since the extra feedback quickly reduces the problem space.

#### Selecting subject population

In order to guarantee that the users we consider interacted with the treatment homework to a significant extent, we restrict our attention to those who completed at least 70 % of the treatment homework and at least 70 % of the common quiz. This cutoff leaves a total of 219 students for unit 10, 205 students for unit 11, and 280 students for unit 12 (cf. a total of 614 students accessed at least one of the treatment problems in all three units). Our results are unlikely to be sensitive to the cutoff, as the majority of students (60–70 %) either completed >90 % of both quiz and homework or <10 % of either.

### Results

#### Comparing DPA and TRD format on developing overall problem solving expertise

##### Time-on-task

We analyzed edX log files to estimate the time students spent on the treatments. The time spent on each problem is estimated by including all the time between loading a problem to students’ last submission to the problem, with a maximum cutoff at 30 min. The median time spent on completing the treatment homework is estimated to be around 5000 s (1.4 h) following this method, with no statistically significant difference among the three different treatments nor among the three different groups found (data not shown). Note that because timing data is highly non-normal, we used the nonparametric Mann-Whitney *U* test to perform pairwise comparison between groups of time-on-task during the homework.

#### Performance of groups on quizzes with traditional problems

Students’ physics problem solving expertise was measured by their performance, i.e., percentage of first attempt correct, on traditional quiz problems. Percentage of first attempt correct was chosen because it reflects the initial performance and shows the largest differences between groups because the majority answered correctly on the first attempt.

The performance of each treatment group on common quizzes is summarized in Table 1. Simple one-way ANOVA reveals no significant difference between the three groups on any of the quizzes. In Table 2, we show the cumulative difference in average quiz performance between the three types of treatment: DPA-D&D, DPA-MC, and TRD. Since we rotate the treatment between the three groups, the overall samples were the same for the three types of treatment (except that some students did not complete all three homework assignments). The only significant difference is between the TRD homework and DPA-MC, while DPA-D&D showed no significant difference with either of the other two.

**Table 1:** Different groups’ treatment and scores on the quiz in three units

	Total score, quiz 10				Total score, quiz 11				Total score, quiz 12			
Treatment	Group	*N*	Mean	SD	Group	*N*	Mean	SD	Group	*N*	Mean	SD
DPA-D&D	A	78	0.61	0.23	C	72	0.47	0.22	B	87	0.59	0.22
DPA-MC	B	71	0.56	0.24	A	64	0.43	0.24	C	103	0.61	0.2
TRD	C	70	0.62	0.23	B	69	0.47	0.23	A	90	0.65	0.18
*p* (ANOVA)	0.23				0.52				0.22

**Table 2:** Difference in quiz score between the three treatment groups

Comparison	Δ*μ* (quiz) (%)	SD (%)	*Z*	*p* (*z* test)
D&D-MC	2.30	2.10	1.11	0.27
MC-TRD	−4.70	2.10	−2.22	0.026^a^
TRD-D&D	2.30	2.10	1.14	0.26

^a^Significant at the 0.05 level

#### Comparing D&D to MC in developing a single skill

The drag-and-drop format did not significantly outperform the MC format when judged by the subsequent traditional problems. This led us to investigate whether the D&D format is superior to the MC format in developing an individual expert skill. We therefore looked at the five DPA sequences that focused on a single basic expert skill, restricting attention to sequences comprised of more than four DPA problems.

**Fig. 4:**
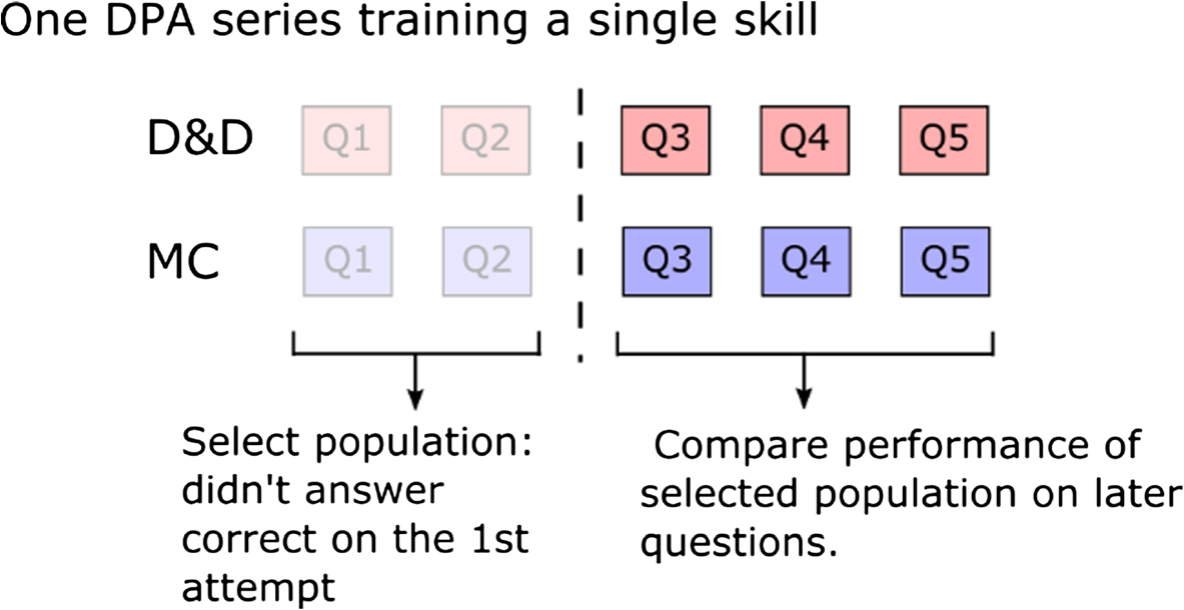
For each DPA sequence containing >4 DPA problems, we used the 1st one or two questions (shown as *2* in the figure) to select the population that needs practice on the skill

The first attempt correct rate on the 1st one or two problems in all five sequences is around 70 %, indicating that the majority of students have previously mastered those skills to some extent. In order to study students who actually need to learn the skills from the activity, we choose to study the 20–30 % of students who did not correctly answer the 1st one or two problems in each sequence on their 1st attempt. We used the 1st two problems for sequences 1 and 2, because they are closely related to each other by design, and should be treated as a unity. The selected population size for each sequence varies from 20 to 40 % of the population that answered >70 % of the given sequence. An example of the analysis is illustrated in Fig. 4.

**Fig. 5:**
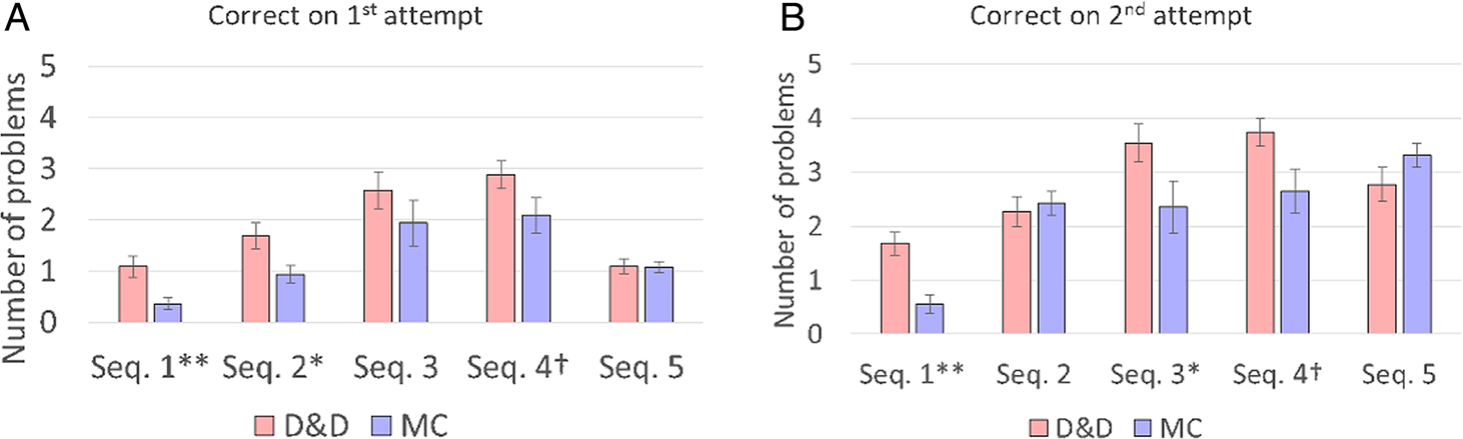
Comparing the average number of correctly answered DPA problems in each DPA sequence. The total number of problems (beyond the 1st one or two that were used to select the sample) in each sequence is shown in Table 3. **a** Average number of correctly answered problems on first attempt. **b** Average number of problems answered correctly on second attempt. **Significant at 0.01 level. *Significant at the 0.05 level. ^†^Significant at the 0.05 level, but population might be dissimilar

We measure the change in individual skills by students’ performance on the subsequent three to four problems in each sequence. As shown in Fig. 5, on three out of five sequences, the D&D group answered significantly more questions correctly on the first attempt than the MC group, and on sequence 3, the D&D group answered more questions correct on the second attempt. Since the distributions are highly non-normal, we used Mann-Whitney *U* test to measure the significance of the difference. As shown in Table 3, a highly significant difference at *p* < 0.01 level is observed for sequence 1 on both first and second attempt correct. Significant difference at *p* < 0.05 level is observed for sequence 2 on first attempt correct and sequences 3 and 4 on second attempt correct. In sequence 2, each MC format question has only three choice items, whereas most questions in other sequences have five or more choice items. Therefore, the extra feedback advantage for MC problem is strongest for sequence 2, which is probably why there is no difference on the second attempt correct. (*t* tests were also performed on the same data with very similar results).

**Table 3:** Difference in average number $\left(\boldsymbol{\Delta} \overline{n}\right)$ of correct answers on subsequent problems per student between D&D and MC format

	Sequence 1	Sequence 2	Sequence 3	Sequence 4	Sequence 5
*n*	3	5	5	5	5
$\overline{n}$(MC, 1st attempt)	0.36	0.94	1.94	2.09	1.08
$\Delta {\overline{n}}_{\mathrm{D}\&\mathrm{D}\hbox{-} \mathrm{M}\mathrm{C},\ 1\ \mathrm{attempt}}$	0.73^a^	0.75^b^	0.64	0.79	0.02
*p* (*U* test)	0.007	0.02	0.25	0.07	0.89
$\overline{n}$(MC, 2 attempts)	1.68	2.27	3.54	3.74	2.77
$\Delta {\overline{n}}_{\mathrm{D}\&\mathrm{D}\hbox{-} \mathrm{M}\mathrm{C},\ 2\ \mathrm{attempts}}$	1.12^a^	−0.15	1.19^b^	1.1^c^	−0.54
*p* (*U* test)	<0.001	0.9	0.03	0.02	0.15

*n* number of problems in each sequence (excluding the 1st one or two problems)

^a^Significant at 0.01 level

^b^Significant at the 0.05 level

^c^Significant at the 0.05 level, but population might be unequal

#### Excluding initial selection bias due to different problem format

Since the initial selection of subjects is based on problems in different formats, these results could arise if students selected in the drag-and-drop groups were stronger but answered the first problem incorrectly because of its unfamiliar problem format. Their subsequent performance would then simply indicate their higher intrinsic ability.

We checked for such selection bias using two different measures. First, if the D&D format selects generally stronger students, then they should have higher physics ability as measured by item response theory (IRT) (Baker 2001; Burton 2004). Secondly, if D&D selects additional stronger students due to format unfamiliarity, then the selected D&D population should be larger than the selected MC population, since random assignment assures similar numbers of weak students in each group.

As shown in Table 4, for sequences 1–3, neither IRT ability nor size of population is significantly different between the two groups at the 0.1 level. For sequences 1 and 2, the D&D population is actually smaller. On sequence 4, the D&D group is somewhat larger than the MC group, and the difference in population is significant at the 0.1 level, whereas in sequence 5, the trend is reversed. On average, there is no difference observed between the two selected populations.

**Table 4:** Comparison of selected population size and IRT ability between D&D and MC groups

	Sequence 1	Sequence 2	Sequence 3	Sequence 4	Sequence 5	Average
% pop. (D&D)	22 %	26 %	26 %	36 %	30 %	28 %
% pop. (MC)	25 %	35 %	18 %	24 %	41 %	29 %
*p* (Fisher)	0.87	0.18	0.23	0.07	0.1	0.8
Mean IRT (D&D)	−0.65	−0.33	−0.54	−0.43	−0.60	−0.45
Mean IRT (MC)	−0.69	−0.66	−0.64	−0.65	−0.18	−0.54
*p* (*t* test)^a^	0.89	0.13	0.82	0.55	0.1	0.43
df	37	53	26	43	46	269
*t*	0.13	1.54	0.23	0.76	−1.44	0.78

^a^Since the distribution is also non-normal, Mann-Whitney *U* test was also performed, with very similar results

### Summary and discussion of learning from DPAs

#### D&D format superior to MC format for DPAs

The drag-and-drop (D&D) format of deliberate practice activities (DPAs) strongly outperforms the multiple choice (MC) format at teaching students to do more DPAs in that particular format. In addition, in an end-of-course survey, 47 % of students rated the D&D format as more intuitive than the MC format, and only 20 % rated the MC format as more intuitive (Chudzicki 2015).

We interpret this as showing that D&D significantly outperforms the MC format in facilitating “rapid” learning of a single expert skill. This conclusion is buttressed by our finding that both the number and the average skill level of the students selected for our study (on the basis of poor performance on the first DPA homework problems) were insignificantly different. This rules out the possibility that students were actually learning to use the D&D format, rather than the desired target skill.

We think that the D&D format is superior mainly because it reduces extraneous cognitive load and increases visual representation. However, there could be other plausible alternative explanations as well. For example, the D&D group has more attempts per problem and students receive less feedback on each attempt, which might force students to “struggle” longer and hence learn more. Also, the new format might simply be more eye-catching to students, leading to better concentration. Further research is required to distinguish between these differences. Of particular importance would be to assess the single skill that the DPAs are supposed to teach using common DPA problems, for example, by breaking down the problem into a few sub-questions.

#### DPAs do not outperform traditional problems as practice for doing traditional problems

DPAs, especially in MC format, are slightly less effective preparation for a quiz containing traditional questions than solving traditional problems for the same amount of time.

It is difficult to believe that our particular implementation of DPA is less effective at teaching the targeted expert skill than solving common physics problems. Therefore, it is likely that there are additional skills involved in overall problem solving expertise; there are a number of equally plausible alternative explanations for this result:

1. The crucial skills that we elected to write DPAs for may not be those that actually result in the students failing to answer the quiz problems correctly. Quite likely, around one fourth of our student population are teachers and might already be fluent with the fairly basic skills that we trained in DPA.
2. Since MOOC students have access to all the learning resources at all times, the quizzes are essentially open-book assessments. Therefore, the assessment is insensitive to any memory benefits of the intervention.

Further work in which the common quiz questions were designed to carefully probe the mastery of the skills targeted by DPAs would seem important for evaluating the outcomes of DPAs.

## Experiment 2: impact of video shooting format on instructor performance and student learning

### Background

High-quality videos by skillful instructors are a hallmark of many successful MOOCs. However, there are also many MOOC videos in which it is apparent that the instructor feels uneasy talking to the camera. While previous studies examined how different video formats impact student engagement (Guo et al. 2014), in this study, we change our viewpoint to explore whether a different video production setting can influence the instructor’s “acting” performance and whether the changes in format together with instructor’s performance will result in detectable differences in student viewing behavior.

Our MOOC 8.MReV contains around 10 “office hour” videos of ~10 min each (but no lecture videos). In each office hour video, the instructor tutors two students on a challenging problem from either the homework or the quiz of that particular unit. This tutoring format is inspired by research on tutorial videos (Muldner et al. 2014), showing that in the right condition, the learning outcome from tutorial videos is comparable to that of live human tutoring. We observed that our tutoring videos often had a relatively slow tempo with frequent pauses while the instructor stops to think or waits for student responses. We hypothesized that this results from the high cognitive load incurred on the instructor by this tutoring setting, in which the instructor has to control multiple aspects of the presentation and interaction with the students while explaining the content at the same time. We call this setup the *instructor-dominant* (ID) setup. To remediate the cognitive overload problem, we changed the video shooting setting and replaced the students in the ID setting with a well-prepared student assistant who shared a lot of the instructor’s load, allowing the instructor to focus on content delivery.

In this experiment, we seek to answer the following research questions:

1. Can we design a video shooting setup that enhances *instructor performance* by reducing cognitive load?
2. Does the instructor’s performance have a detectable impact on students’ *video watching behavior*?
3. Does better instructor performance lead to detectable improvement in students’ *learning outcome*?

### Methods

#### Video shooting setups

##### ID video setting

**Fig. 6:**
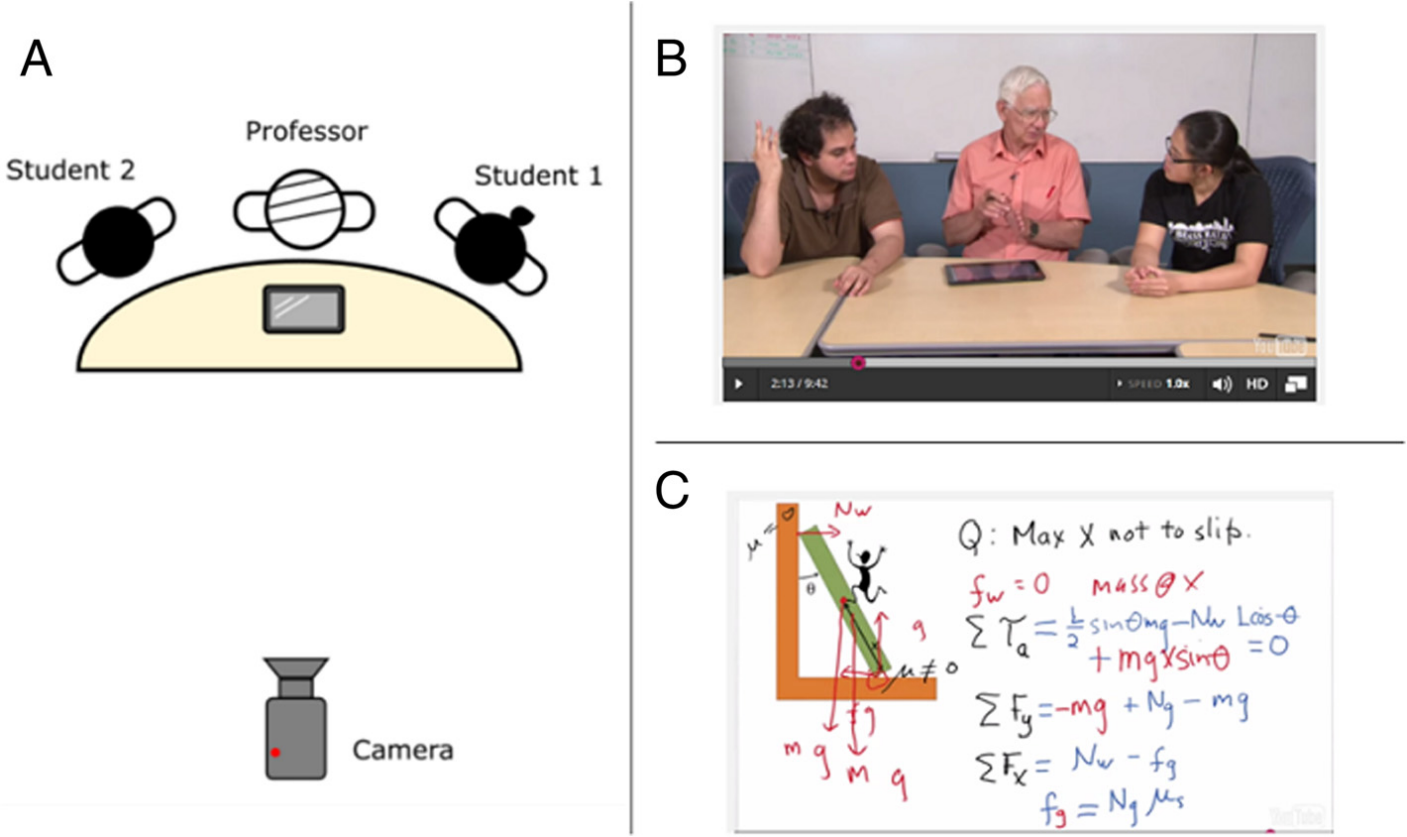
ID video shooting setup. **a** Schematic drawing of the setup. **b** Screen shot of ID format video. **c** Example of tablet capture

The original ID format videos were shot during the 2013 run of 8.MReV. In this setting (Fig. 6), both the instructor and students sit at a table facing the camera. A tablet computer with stylus input was placed in front of the instructor, on which the problem was displayed. The instructor tutors the two students on solving the problem and writes the solution to the problem on the tablet. The tablet capture, which resembles the Khan Academy-style video, was later integrated into the camera footage as part of the video.

##### Assisted tutoring (AT) video setting

**Fig. 7:**
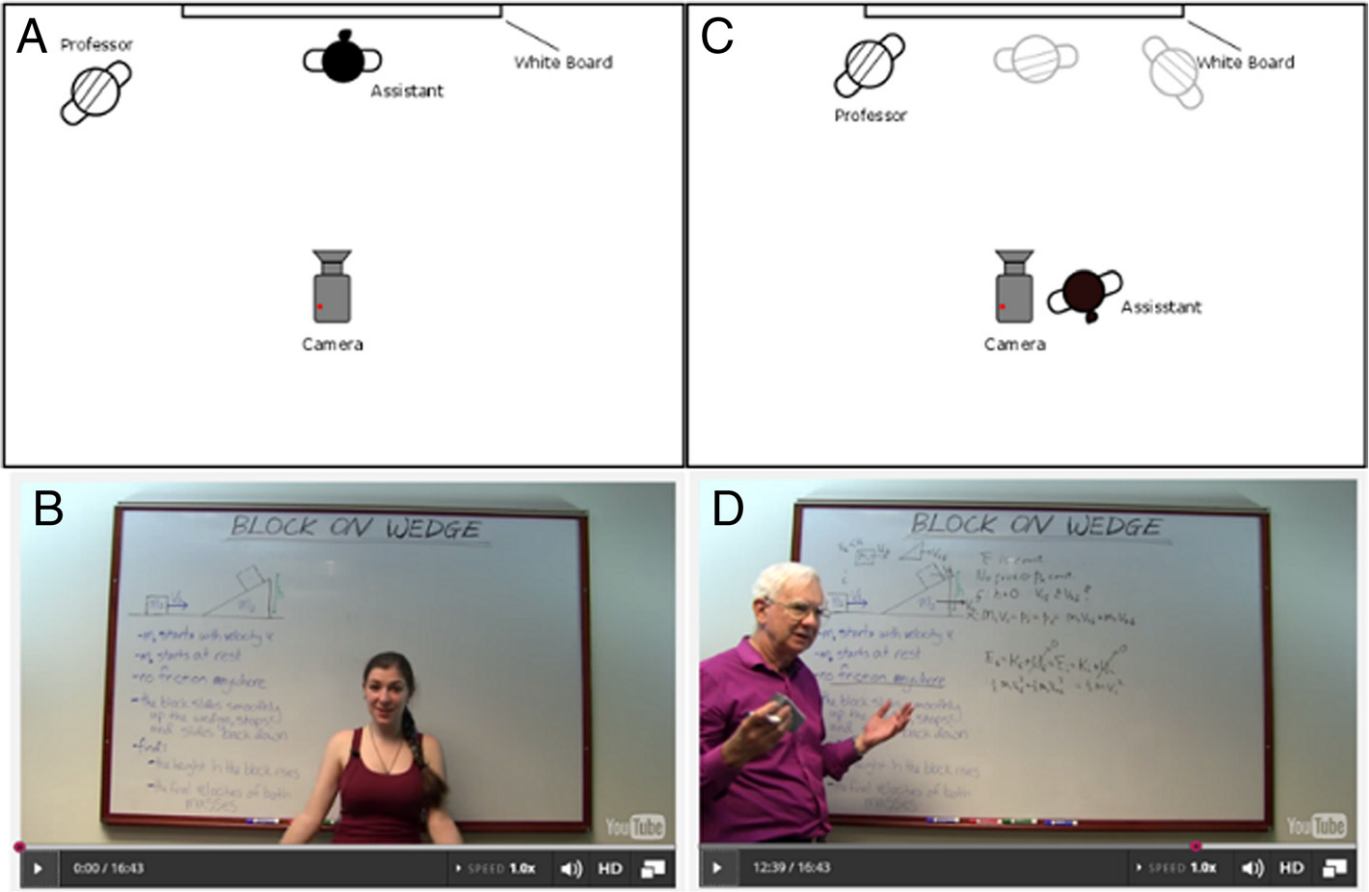
AT video shooting setting. **a**, **b** A teaching assistant introduces the problem at the beginning of the video. **c**, **d** The instructor comes into the scene, and the assistant retreats beside the camera

In 2014, we re-shot four of the office hour videos with the same instructor in AT setting. In this setting (Fig. 7), a student assistant first outlines the problem on a whiteboard and invites the instructor to explain it. As the instructor enters the scene and starts to explain the problem, the assistant stands beside the camera (out of the scene) serving as the audience. For emphasizing a key point, or where the assistant feels the instructor should elaborate his explanation, the assistant will raise that point by asking the instructor a question. Before the shooting of each video, the assistant prepares a list of potential questions that could be asked during the video shooting.

#### Experiment setup

**Fig. 8:**
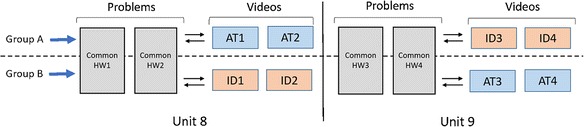
Experiment design of experiment 2

The experiment was conducted in the homework section of units 8 and 9 of the 2014 MOOC, 8MReVx (Fig. 8). Each unit contains two videos shot in both settings placed at the end of the homework sequence. Each video explains one problem in the homework. Upon accessing one of those two homework problems, students are provided with a link pointing to the corresponding video, together with a text prompt encouraging them to attempt the problem at least once before watching the video. On the page containing the videos, there is another link that points students back to the problem, allowing students to go back and forth between the video and the problems. Separating the videos from the problems allows us to easily track the order in which students accessed the videos.

The student population was randomly assigned to two groups, A and B. Those assigned to group A will be presented with two ID videos in unit 8 and two AT videos in unit 9, whereas the treatments were reversed for group B.

### Results

#### Instructor performance and speech speed

Experientially, the overall presentation of the instructor in the AT setting is noticeably more fluent than in the ID setting. As a first-order measurement of fluency, we measured words per minute (wpm) for three of the four videos for which verbal transcripts were available (transcript for video 2 ID format was lost due to file corruption). The wpm is simply estimated by dividing the total length of the transcript by the length of the video (Guo et al. 2014). As shown in Table 5, in all three videos, the wpm in the AT setting is higher than that in the ID setting and the difference is larger for videos 3 and 4. Noticeably, the lengths of the two types of videos were not very different, which indicates that the instructor conveyed more information in the AT format than the ID format.

**Table 5:** Comparison between two different formats of videos. Mean total viewing time for each video is reported as the percentage of total video length, for meaningful comparison between videos of different lengths

Videos	Time (s)	wpm	*N* (attempted problem)	*N* (watched video)	Mean total viewing time	% of students with long views	% of students with correct final answer
AT1	1004	125	318	36	75 %	25 %	74 %
ID1	1187	118	308	39	96 %	26 %	66 %
					*p* = 0.02 (*U* test)	*p* = 0.94 (chi sq.)	*p* = 0.61 (chi sq.)
AT2	659	N/A	294	113	88 %	41 %	93 %
ID2	583	N/A	290	105	108 %	44 %	83 %
					*p* = 0.05 (*U* test)	*p* = 0.64 (chi sq.)	*p* = 0.04 (chi sq.)
AT3	731	142	223	67	101 %	45 %	82 %
ID3	943	113	206	75	79 %	27 %	84 %
					*p* = 0.006 (*U* test)	*p* = 0.02 (chi sq.)	*p* = 0.8 (chi sq.)
AT4	377	145	212	39	79 %	46 %	84 %
ID4	423	122	195	46	83 %	28 %	98 %
					*p* = 0.42 (*U* test)	*p* = 0.09 (chi sq.)	*p* = 0.02 (chi sq.)

#### Student population

Around 200–300 students attempted the two homework problems in each unit. Among those students, less than 30 % accessed the videos before submitting their last attempt on the problem. Furthermore, as shown in Table 5, while the number of students who attempted the problem stayed relatively constant within each unit, the fraction of students accessing the videos fluctuated much more between problems. This is somewhat surprising to us as instructors; the videos contain detailed explanation to the problems, and the first attempt correct rate for non-video viewers ranges from 30 to 70 %. For the rest of the data analysis, we will concentrate on those students who accessed the video before their last attempt on the problem.

#### Change in students’ viewing behavior

The total viewing time showed an inconsistent trend between the AT and the ID videos: for the first two videos, students spent more time watching the ID version, whereas for the third video, students spent more time watching the AT version. Since the distribution of time data is highly non-normal, we used Mann-Whitney *U* test as a test of significance. For continuous random variables such as time-on-task, the Mann-Whitney *U* test can be interpreted as testing for a difference in median if the distributions are similar (Sheskin 2004). Since a significant fraction of students watched close to 100 % of the video in both cases, the median watch percentages are the same in each case (100 %). Therefore, we report the mean percentage to better reflect the trend of differences.

As an indicator of students’ level of engagement with the video, we calculated the percentage of students with at least one long view event. Using view length as a measure of engagement has been used by previous MOOC video research (Guo et al. 2014). We defined a “long view” event as one unstopped play event that is longer than 70 % of the total video time. The hypothesis is that for less engaging videos, students are more likely to skip part of the video and search for information that is useful to them, generating more pause and search events and shorter view events. On the other hand, the mean viewing time, a measure of total engagement, differed significantly on three of the four videos; however, the direction varied among the videos so no global conclusion seems warranted.

While videos 1 and 2 showed little difference, on videos 3 and 4, the AT version resulted in more students with long views. The difference is statistically significant for video 3 but not for video 4 due to a smaller sample size. Notably, videos 3 and 4 are also the ones where the speech speed difference between AT and ID is the largest.

#### Changes in learning outcome

Students’ learning outcome is measured by their performance on the problem. Notice that the first attempt is not a valid measurement in this case, because most students submitted their first attempt before watching the video. Since all subjects watched the video before their last attempt on the problem, we used their last attempt correct rate as a measure of learning from the video.

Again, as shown in Table 5, we did not observe any consistent trend favoring one video format over the other among the four instances. For video 2, students watching the AT version outperformed students watching the ID version by 10 %, whereas on video 4, the trend was flipped.

### Discussion of video format experiment

Our primary finding was that in the assisted tutoring (AT) format made the instructor produced more fluent explanations with fewer pauses than in the ID setting, presumably because of reduced cognitive load.

When it comes to students’ video watching behavior, the AT format leads to increased engagement, as measured by the percentage of students with one uninterrupted long view. This result is in line with the general conclusion from an earlier study (Guo et al. 2014) that faster speech leads to better engagement. However, their detailed findings for the particular range of speech speeds (100–150 wpm) and video length (9–12 min) indicated a lower engagement level for higher speech speed.

A key factor in video watching is whether students learn from them and whether high engagement is accompanied by greater learning. Unfortunately, most MOOC video studies made no attempt to determine whether students learned from the videos. We did try to measure learning in the current experiment, yet no significant difference was observed for students’ performance on homework problems, which we suspect is partly due to the ceiling effect (most got it correct irrespective of watching the video) as well as the small sample size of those who watched the video. In particular, we did not test for transfer of learning from the video in this experiment, which could be more sensitive to the difference in engagement.

A noteworthy observation is that, although the videos contain detailed explanation to the problem, only a small fraction of students chose to watch the video, and this fraction is different for different problems. Furthermore, students who did not watch the video were far from perfect on their first attempt of the problem. This suggests that video watching is a deliberate decision made by MOOC students, and most of them prefer to figure out the problem on their own as much as they can. Upon closer inspection, we also found that over half of the students who watched the videos followed our suggestion and made their first attempt before watching the video. These behaviors suggest that MOOC students are highly motivated learners, whose behavior is not representative of typical high school or college students.

## General discussion: the strengths and challenges of MOOC AB experiment

The two MOOC AB experiments we report here cover two of the most important topics in MOOC research in Science, Technology, Engineering, and Math (STEM): developing effective pedagogies for teaching expert problem solving and learning from videos.

Our first experiment on deliberate practice activities shows that properly designed drag-and-drop problems seem to develop individual expert skills significantly better than multiple choice format. However, our deliberate practice activities were no better than traditional homework practice in improving student performance on solving traditional physics problems. Thus, learning the particular expert skills that we focused in the DPAs does not transfer to solving the traditional problems. This could have many explanations including that we did not pick the critical skill for solving the traditional problems, that our learning format does not induce transfer, or even that our students use novice heuristic methods to solve the traditional problems so that the possession of the expert skill is of no relevance.

The second experiment shows that the new video shooting setting improves the instructor’s performance by reducing cognitive load and improves student engagement. However, its impact on learning outcomes was not significant.

Our first attempts at conducting and analyzing learning from AB experiments in a MOOC environment did not result in eye opening discovery or solid conclusions. This is not completely unexpected, as the majority of online AB testing shows no effect (Kohavi and Longbotham 2015). However, in each case, the size of the samples was sufficient to reveal some significant effect: the more rapid learning occurring from drag-and-drop DPAs (relative to multiple choice) and the importance of reducing the cognitive load on the instructor for generating more engagement.

The strength of MOOC AB experiments is obvious: The flexibility of the online platform allows researchers to easily conduct experiments with multiple repetitions and large sample size. Rich click stream data provides ample opportunity for in-depth data mining and analysis, such as students’ video viewing pattern, time spent on solving problems, or the order in which students access different contents in the course. Notably, the significant results obtained in both experiments were on selected subsamples of the total population, which resulted in significantly smaller samples than one would have expected from the large MOOC registration numbers. On the other hand, this selection may eliminate many of the extraneous students in MOOCs, e.g., the teachers of that subject (who are already experts) and those students who used a second account to obtain the correct answers to problems.

Finally, we discuss the many challenges that MOOC AB experiments face along three axes: the student, the assessment, and the data.

On the student axis, the MOOC student population has great difference in background and objectives and displays typically four times the variance in skill (Colvin et al. 2014) as students who have been filtered by selection to a particular class in a particular college. On the one hand, we have a lot of highly self-motivated and highly skilled students (Ho et al. 2015, 2014). In experiment 1, for example, about 60–70 % of students come in with good initial knowledge on many of the expert skills that we designed DPAs to train. On the other hand, our group recently detected the existence of “cheaters” in the course who use a second account to harvest correct answers and input them into their main account (Alexandron et al. 2015).

The implication is that whatever instructional method (and the level of its content) we try to evaluate will likely make a difference to only a subset of the entire MOOC population. Therefore, if we simply look at the entire population without proper selection, it is likely that we will be overwhelmed by background noise from students for whom this intervention is ineffective or unwarranted.

A possible solution to this problem is to include some kind of “pre-test” as part of the experiment, which will allow the researcher to select the populations that are most likely to be sensitive to the different treatments. For example, the D&D vs. MC comparison was only significant after we selected the subpopulations that are initially weak on the given skill.

On the assessment axis, the validity of MOOC assessments is challenged by the fact that essentially all MOOC quizzes are open book. Moreover, we have little to no control over the time and order in which students complete the assessments or if they complete them at all (particularly since passing in most MOOCs is at the 60 % level). This poses a significant challenge to the inference of causal relations between students’ learning activity and assessment performance. For example, in both experiments, students could have learned how to solve the assessment problems from other parts of the course or even from a different website or a book. Highly skilled students could even have completed the assessment and the intervention in reverse order.

One potential strategy for dealing with this challenge is to carefully analyze students’ log data and determine the order of their activities. For example, in experiment 2, we only selected those students who watched the video before submitting their last attempt on the assessment problem. However, this type of analysis can often be expensive and time consuming and is powerless to student activities happening outside of the MOOC platform, such as reading a book or visiting another website. Another strategy is to design the learning process itself as the assessment, such as in the D&D vs. MC study, where students’ change in performance during the learning process is used as the assessment metric. This is because students are more likely to complete the whole training process in one setting and have less incentive to search for outside help.

Yet perhaps the best solution to this problem is for future MOOC platforms to provide more control over students’ behavior, such as requiring students to complete the intervention before they can “unlock” the assessment or providing the ability to administer time-limited tests.

Finally, the richness of MOOC data also presents challenges for analysis. For example, performance on any assessment problem can be measured by first attempt correct rate, last attempt correct rate, average number of attempts used, and time spent on each attempt. On what basis should we choose the most appropriate measurement for a given analysis? How do we deal with the learning value of a few failed attempts prior to consulting some instructional resource? For the experiments presented here, most of those decisions are based more on intuition plus trial and error to find good signal-to-noise ratios, rather than evidence-based research. Understanding and extracting the information carried in different types of data is certainly a much needed future direction in MOOC analytics. In addition, MOOC data—especially time data—have highly non-normal and unique distribution, which requires new mathematical tools and statistical models to analyze (Lamb et al. 2015).

In summary, while AB experiments are already a promising research tool in MOOCs, much effort is needed to address the various challenges that MOOCs present in order to make it an effective tool for education research.

## Abbreviations

AT: assisted tutoring
D&D: drag and drop
DPA: deliberate practice activities
ID: instructor dominant
MC: multiple choice
MOOC: massive open online course
TRD: traditional
